# Biochemical tolerance during low dose propylene glycol exposure in neonates: A formulation-controlled evaluation

**DOI:** 10.1186/1560-8115-20-5

**Published:** 2012-07-19

**Authors:** Aida Kulo, Anne Smits, Gunnar Naulaers, Jan de Hoon, Karel Allegaert

**Affiliations:** 1 Center for Clinical Pharmacology, University Hospitals Leuven, Leuven, Belgium; 2 Institute of Pharmacology, Clinical Pharmacology and Toxicology, Faculty of Medicine, University of Sarajevo, Sarajevo, Bosnia Herzegovina; 3 Neonatal Intensive Care Unit, University Hospitals Leuven, Leuven, Belgium; 4 Neonatal Intensive Care Unit, University Hospitals Leuven, Herestraat 49, Leuven, 3000, Belgium

**Keywords:** Propylene glycol, Solvent, Excipient, Formulation, Safety, Tolerance

## Abstract

**Background and purpose of the study:**

Propylene glycol (PG) is a frequently co-administered solvent in formulations administered to neonates, but reports on its (in)tolerance are limited. We aimed to report on renal, metabolic and hepatic tolerance before, during and following intravenous (iv) PG-paracetamol exposure and compared these data with similar datasets reported in literature on neonates exposed to PG without paracetamol or paracetamol without PG.

**Methods:**

Renal (diuresis, creatinemia, sodium), metabolic (Base Excess, Anion Gap, lactate, bicarbonate) and hepatic (liver enzymes, bilirubinemia) indicators before, during and following iv paracetamol-PG exposure in neonates as included in the PARANEO (*para*cetamol in *neo*nates) study (intra-individual trends, ANOVA) were collected and analysed. Comparison with observations collected in cases exposed to either iv phenobarbital-PG or iv paracetamol-mannitol (inter-individual comparison, Mann Whitney-*U* test) were made.

**Results:**

PG exposure (median 34.1 mg/kg/24 h) did not affect postnatal renal, metabolic and hepatic adaptations in 60 cases exposed to paracetamol-PG. These indicators were similar when compared to 29 cases exposed to phenobarbital-PG or 172 cases exposed to paracetamol-mannitol.

**Major conclusion:**

Based on observations in 89 neonates, low dose PG exposure was tolerated well. Studies on PG pharmacokinetics and its covariates are needed to estimate the upper level of PG tolerance in neonates.

## Introduction

Propylene glycol (1,2 propanediol, PG) is a clear, colourless, odourless, water-soluble alcohol. Physically, it is similar to ethylene glycol but PG is claimed to be less toxic [1,2]. However, PG exposure can cause lactic acidosis, may result in an increase in Anion Gap or Osmolar Gap, hyponatremia or hepatic dysfunction. Other side effects such as hemolysis, mental status changes or renal toxicity (e.g. renal tubular acidosis, acute tubular necrosis resulting in increased serum creatinine and oliguria) were reported as manifestations of PG toxicity [1-3]. Most of these reports relate to continuous intravenous (iv) administration of benzodiazepines (e.g. lorazepam) containing PG as solvent in adult intensive care setting, resulting in PG exposure above 200 g/day or 2 g/kg/day [4].

Based on the current evidence, PG toxicity relates to its accumulation, and therefore is driven by the extent of exposure and the individual elimination capacity [5]. Median PG clearance following exposure to 3–15 g PG/m^2^ in adults was 15.9 L/h, the estimated elimination half life 2–5 hours. About 45% was eliminated by renal route, 55% underwent hepatic metabolisation through lactate and pyruvate [3]. Consequently, impaired hepatic or renal clearance increases the likelihood of PG accumulation and subsequent toxicity. By virtue of their nature, infants combine limited renal elimination with limited hepatic metabolism.

Data on PG exposure in neonates have been reported by Shehab *et al.*, Chicella *et al.*, MacDonald *et al.* and by the Food and Drug Administration (FDA) [6-10]. Shehab *et al.* described PG exposure (median 426 mg/kg/day) in 82 neonates to raise awareness on PG exposure in neonates [6]. Chicella *et al.* published on PG accumulation associated with continuous infusion of lorazepam in 11 infants (age range 1–15 months) [7]. The authors hereby described PG accumulation during continuous infusion without clinical and laboratory abnormalities (Osmolar Gap, lactate). In contrast, toxicity related to PG has been reported in preterm neonates following exposure of up to 3000 mg/day for at least 5 days. This toxicity was clinical (seizures) and biochemical (hyperosmolarity, lactic acidosis, raised serum creatinine, bilirubin) [8,9]. Similarly, there is a recent FDA drug safety communication on serious health problems in preterm neonates exposed to an oral solution of lopinavir/ritonavir (Kaletra®, Abbott Laboratories Illinois, United States), containing ethanol (42.4% v/v) and PG (15.3% w/v) [10].

In the current paper, we focus on aspects of PG tolerance/toxicity by reporting on a further extended prospectively collected dataset [11] on renal, metabolic and hepatic tolerance following iv paracetamol-PG exposure in 60 neonates included in the PARANEO (*para*cetamol in *neo*nates) study [12]. We compared these observations during paracetamol-PG exposure with similar observations in neonates exposed to formulations containing PG without paracetamol [11] or paracetamol without PG [13,14].

## Methods

### PARANEO study, paracetamol-PG study

The study was conducted in Leuven NICU following approval by the local ethical board of the University Hospitals Leuven (B-32220084836) and study registration (EUdraCT 2009-011243-39, http://www.clinicaltrials.gov). Neonates were included after informed written parental consent. The decision to prescribe a source of intravenous PG, either paracetamol-PG or another PG containing formulation, was made by the attending physician and based on the clinical needs.

The Leuven Neonatal Intensive Care Unit has 35 intensive care beds and another 8 high care beds. The clinical part of the study was between September 2009 and December 2010. Observations were collected during repeated iv paracetamol-PG (Paracetamol Synthetica®, Treviso, Italy, 0.8 mg PG/mg paracetamol) administration in 60 neonates. Indications for iv paracetamol administration were medical (traumatic delivery, necrotizing enterocolitis, prostaglandin E_2_ administration, fever) or postoperative (e.g. cardiac, thoracic, abdominal). The dosing regimen comprised a loading dose (20 mg/kg paracetamol), followed by a maintenance dose of 5 mg/kg, 7.5 mg/kg or 10 mg/kg every 6 hours for extreme preterm (i.e. born before 32 weeks gestational age), preterm (<37 weeks gestational age) and term (≥ 37 weeks gestational age) neonates and was maintained for 48 h [12]. Based on the formulation used (0.8 mg PG/mg paracetamol), this resulted in PG co-administration of 16 mg/kg, 4, 6 and 8 mg/kg.

Biochemical indicators of PG related toxicity were based on biochemical indicators earlier reported in literature and relate to renal [creatinaemia, plasma sodium, diuresis (ml/kg/h)], metabolic [Base Excess (BE), Anion Gap (AG), lactate, bicarbonate] or hepatic [aspartate transaminase (AST), alanine transaminase (ALT), direct bilirubinemia] disturbances [11]. To facilitate sampling, and to make observations throughout time comparable, only patients with an arterial line were included. All observations available in a time interval of 48 h before the first administration up to 48 h after the last PG exposure were collected.

Sampling and analysis of samples was performed based on the prescription of the attending physician since routinely collected biochemical data were used to evaluate PG (in) tolerance [11].

### Other cohorts to compare with

Renal and metabolic observations collected in this group of 60 neonates exposed to paracetamol-PG were compared with similar observations prospectively collected in 29 neonates exposed to other sources of PG [11]. Luminal© Injektionslösung, (Desitin Arzneimittel, Hamburg, Germany) 700 mg PG/200 mg Phenobarbital, Lanoxin© (GlaxoSmithKline, Genval, Belgium), 828 mg PG/0.5 mg digoxin and Diphantoine iv^©^ (Kela Pharma, Sint Niklaas, Belgium), 2 000 mg PG/250 mg phenytoin were identified as sources of intravenous PG in the Leuven NICU [11] (B-32220084836).

Similarly, observations on hepatic and renal tolerance during and following paracetamol-PG were compared with earlier reported observations on hepatic and renal tolerance following paracetamol-mannitol (Perfusalgan®, Bristol Myers Squibb, Braine L’Alleud, Belgium) exposure in 149 and 23 cases respectively [13,14]. All paracetamol exposed cases were treated with a similar amount of paracetamol (20–40 mg/kg/24 h after an initial loading dose of 20 mg/kg).

### Statistics and analysis

Clinical characteristics and indicators of renal, metabolic or hepatic disturbances before, during and after paracetamol-PG exposure were described by median, standard deviation and range. Intra-individual trends of biochemical indicators were analysed using ANOVA (trend analysis, following logarithmic conversion if data were not normally distributed) and paired analysis (before, during and following). Observations (before, during, following exposure) collected in paracetamol-PG cases were compared (unpaired Mann Whitney-*U* test) with observations collected in cases exposed to other PG sources [11] or to paracetamol-mannitol [13,14]. (MedCalc®, Mariakerke, Belgium) was used. A p-value < 0.05 was considered significant.

## Results

The clinical characteristics in 60 neonates included in the PARANEO study are provided in Table 1 and reflect the heterogeneous population of extreme preterm neonates (< 32 weeks gestational age) to term neonates with congenital malformations exposed to PG. In Table 2, observations as collected in PARANEO cases (colomn 1) were compared to observations reported in cases exposed to paracetamol-mannitol (column 2). Column 3 provides the same type of observations but compared to observations in 29 neonates exposed to other PG sources in the same neonatal unit and in the same time interval. Median PG exposure for both cohorts (n = 60 + 29) was 145.6 (17.5-591.5) mg, 62 (14.8-284) mg/kg or 34.1 (14–252) mg/kg/24 h respectively. There were no significant differences in PG exposure or clinical characteristics between both PG exposed cohorts.

**Table 1:** Clinical characteristics of 60 neonates included in the PARANEO study

***clinical characteristics***	***median***	***standard deviation, range***
*Gestational age (wks)*	37	4.1, 25 – 42
*Postnatal age (days)*	3	6.7, 1 – 28
*early neonatal life (<7d)*	44/60	
*Birth weight (kg)*	2.71	0.91, 0.6 – 4.3
*VLBW (<1500 g)*	10/60	
*Current weight (kg)*	2.7	0.92, 0.606 – 4.3
*PG exposure, (total mg)*	166	96, 21.6 – 466
*PG exposure (mg/kg/24 h)*	33.35	38, 14.8 - 252

(Data reported by median, standard deviation and range).

(PG = propylene glycol; VLBW = very low birth weight, < 1500 g at birth, at inclusion 9/60).

**Table 2:** Indicators of propylene glycol (PG) (in) tolerance are summarized in 60 cases exposed to paracetamol-PG (PARANEO study), to paracetamol-mannitol or to other sources of PG (n = 29)

	**paracetamol-PG**			**paracetamol-mannitol**			**other sources of PG**		
	**ref**[12]			**ref**[13,14]			**ref**[11]		
	**before**	**during**	**following**	**before**	**during**	**following**	**before**	**during**	**following**
**Renal indicators**									
Diuresis (ml/kg/h)	3.7, SD 1.2	4.7,SD 1.3	4.8, SD 1.5	n.a.	4.8, SD 1.5	n.a.	3.7, SD 1.5	5.2, SD 1.6	4.8, SD 1.2
	(1.2-7.0)	(1.9-8.9)	(2.1-9.9)				(1.1-7.5)	(2.5-11)	(1.8-9.5)
Creatinemia(mg/dl)	0.62, SD 0.19	0.56, SD 0.18	0.49, SD 0.16	n.a.	0.57, SD 0.19	n.a.	0.62, SD 0.26	0.52, SD 0.28	0.66, SD 0.62
	(0.25-1.05)	(0.21-1.05)	(0.24-1.02)				(0.19-1.36)	(0.22-1.32)	(0.25-2.9)
Sodium (mEq/l)	139, SD 4.9	139, SD 3.8	139, SD 4.1	n.a.	n.a.	n.a.	138.5, SD 4.9	141, SD 6.6	140, SD 5.3
	(127–154)	(129–148)	(123–148)				(128–151)	(125.8-154.8)	(127–151.3)
**Metabolic indicators**									
Base Excess									
	−3.7, SD 4.0	−3.0, SD 3.5	−1.4, SD 4.3	n.a.	n.a.	n.a.	−3.6, SD 3.9	−2.8, SD 3.4	−4.0, SD 4.6
Anion Gap	(−14.5-10.1)	(−14.4-8.6)	(−16.7-7)				(−21.6-3.2)	(−13.5-7)	(−16.9-7.8)
	13.6, SD 3.1	12.8, SD 2.4	12.4, SD 2.3	n.a.	n.a.	n.a.	14.1, SD 4.3	11.1, SD 3.2	11.6, SD 2.6
Lactate (mg/l)	(7.2-21.7)	(7.2-20.9)	(7.9-18.9)				(8.4-27.3)	(6.3-22.5)	(7–21.3)
	1.1, SD 0.8	1, SD 0.9	0.9, SD 0.65	n.a.	n.a.	n.a.	2, SD 2.8	1.6, SD 0.8	1.0, SD 0.4
Bicarbonate (mEq/l)	(0.4-5.2)	(0.4-8.5)	(0.4-6.4)				(0.6-16)	(0.4-6.2)	(0.4-2.9)
	21.8, SD 3.8	22, SD 3.5	22.7, SD 3.8				21.7, SD 3.6	22.9, SD 3.5	21.7, SD 4.2
	(11.2-35.9)	(11.8-34.4)	(8.3-33.5)	n.a.	n.a.	n.a.	(7–29.6)	(15.1-31.5)	(12.1-35)
**Hepatic indicators**									
AST (IU/l)	33, SD 57	35, SD 35	30, SD 35	34, SD 68	30, SD 41	26, SD 38	n.a.	n.a.	n.a.
	(7–256)	(13–229)	(14–196)	(12–1 068)	(10–376)	(8–198)			
ALT (IU/l)	9, SD 58	11, SD 53	12, SD 88	13, SD 32	13, SD 32	13, SD 28	n.a.	n.a.	n.a.
	(4–411)	(4–318)	(5–489)		(4–216)	(4–117)			
Direct bilirubinemia (mg/dl)	0.43, SD 0.4	0.39, SD 0.4	0.47, SD 0.5	(4–216)	n.a.	n.a.	n.a.	n.a.	n.a.
	(0.01-2.39)	(0.01-2.2)	(0.01-2.7)	n.a.					

Observations collected before (−48 h until start), during, and after (until +48 h after the last administration are reported. Data are provided by median, standard deviation (SD) and range (n.a = not available).

*Renal indicators* of potential paracetamol-PG toxicity (Table 2, renal indicators) were diuresis (291 observations), creatinemia (295 observations) and sodium (322 observations). Using ANOVA, there is a progressive increase in diuresis (3.7 before to 4.7 and 4.8 ml/kg/h during and following paracetamol-PG exposure respectively, p < 0.05). When we focus on diuresis (n = 105) during PG exposure 48 h time interval, there is not a significant change in diuresis.

There are no significant differences between cases exposed to paracetamol-PG compared to other sources of PG (n = 139, median values 3.7, 5.2 and 4.8 ml/kg/day) and are similar to the 4.8 ml/kg/h reported in neonates exposed to paracetamol-mannitol. Similarly, there is a progressive, significant decrease in serum creatinine when observations collected before, PG exposure were compared with observations collected during or following exposure, irrespective of the PG source (column 3, p < 0.05). There is no significant trend in serum creatinine during exposure. Finally, serum creatinine during paracetamol-PG exposure is similar to paracetamol-mannitol cases (colomn 2).

*Metabolic indicators* of paracetamol-PG toxicity were Base Excess (987 observations), Anion Gap (292 observations), lactate (1041 observations) and bicarbonate (1361 observations). Using ANOVA, there is a progressive decrease - reflecting normal postnatal adaptations - in Base Excess, Anion Gap, lactate and bicarbonate (Table 2, all at least p < 0.05). Based on 606, 129, 611 and 767 observations to compare with cases exposed to other PG sources, there were no significant differences between both cohorts (Table 2, colomn 1 and 3).

Finally, *hepatic indicators* of potential paracetamol-PG toxicity were AST (136 observations), ALT (130 observations) and direct bilirubinemia (191 observations). Using ANOVA, no significant changes throughout consecutive time intervals were observed. When compared to a published dataset on hepatic tolerance before, during and following paracetamol-mannitol exposure, no significant differences were observed (Table 2, colomn 1 and 2).

## Discussion

Embedded in a prospective study on iv paracetamol disposition in 60 neonates, we documented that a median PG exposure of 34.1 (range 14–252) mg/kg/24 h for 2 days did not affect normal postnatal renal, metabolic and hepatic adaptations. These observations were similar to cohorts exposed to other sources of either iv PG or iv paracetamol.

Whittaker *et al.* refocused on the exposure to additives in medications used in describing the presence of several solvents - including PG - in drugs administered to neonates in the absence of any data on the safety level of exposure [14]. Similar to the established use of off-label drugs, we have to be aware that we are in a setting of established (co)administration of solvents in neonates [6,7,15]. PG toxicity relates to accumulation, and is therefore driven by exposure and elimination capacity [1-4]. Although conclusions on tolerance can only be based on biomarkers initially reported in adults and only focus on short term outcome, the current observations suggest that there is a lower limit of safe short term exposure to PG in neonates (< 35 mg/kg/24 h) similar to the spectrum concept of adult safe exposure level, claimed to be about 1000 mg/kg/24 h [5].

Information on PG pharmacokinetics in neonates is needed to estimate the level of PG tolerance in neonates. At present, the data on PG pharmacokinetics in neonates are very limited. The group of MacDonald estimated a PG serum elimination half life in preterm neonates (all < 1.5 kg) to be 10.8 to 30.5 h [8,9]. To compare observations in preterm neonates with more mature neonates, we recalculated PG pharmacokinetics in a dataset of 22 PG plasma (median PG concentration 31.9, range 15–46 mg/l) samples collected in a cohort of 6 (near)term neonates (median weight 3.08 kg, median postmenstrual age 38 weeks) [16]. Assuming a one compartment model, with zero order input and first order output, the calculated PG elimination half life is 8.88 h, PG clearance is 0.0683 l/h. The differences in elimination half life at least suggest that age or weight will be one of these covariates [17,18].

In conclusion, based on renal, metabolic and hepatic indicators of PG toxicity collected in 89 neonates, a median exposure of 34.1 mg/kg/24 h, is tolerated well. Studies on PG pharmacokinetics and its covariates are needed to estimate the upper level of PG tolerance in neonates.

## Competing interest

There are no other conflicts of interest related to this publication.

## Authors’ contribution

All authors contributed to the concept and design, analysis and interpretation of data, drafting and revising and final approval. KA was responsible for the study registration, AK and AS for the data acquisition.
